# Errata

**Published:** 1853-01

**Authors:** 


*Errata.*—Unfortunately many typographical errors crept into my article in the April number.

On page 425, 6th line from bottom, after the word diseased, read *bone and.*

On page 427, 11th line from bottom, strike out the quotation marks after the word *Although.*

Page 428, 10th line from bottom, for *even*, read *ever.*

Page 430, 20th line from top, for *ridiculous*, read *solicitous.*

Page 431, 11th line from top, for *any*, read *by.*

Page 435, 17th line from top, let the sentence end with the word *effect*, and place a comma after the word *cuticle.* 10th line from bottom, for *they are*, read *it is.*

Page 436, 3d line from top, before the word *tooth*, insert *safety of the.*

Page 437, 6th line from the top, after the word crepitus, insert *sound.*

The errors of punctuation are "too numerous to mention."

